# Cooperative Breeding and Long-Distance Dispersal: A Test Using Vagrant Records

**DOI:** 10.1371/journal.pone.0058624

**Published:** 2013-03-14

**Authors:** Caroline L. Rusk, Eric L. Walters, Walter D. Koenig

**Affiliations:** 1 Cornell Lab of Ornithology, Ithaca, New York, United States of America; 2 Department of Biological Sciences, Old Dominion University, Norfolk, Virginia, United States of America; 3 Department of Neurobiology and Behavior, Cornell University, Ithaca, New York, United States of America; CNRS, Université de Bourgogne, France

## Abstract

Cooperative breeding is generally associated with increased philopatry and sedentariness, presumably because short-distance dispersal facilitates the maintenance of kin groups. There are, however, few data on long-distance dispersal in cooperative breeders—the variable likely to be important for genetic diversification and speciation. We tested the hypothesis that cooperative breeders are less likely to engage in long-distance dispersal events by comparing records of vagrants outside their normal geographic range for matched pairs (cooperatively vs. non-cooperatively breeding) of North American species of birds. Results failed to support the hypothesis of reduced long-distance dispersal among cooperative breeders. Thus, our results counter the conclusion that the lower rate of speciation among cooperative breeding taxa found in recent analyses is a consequence of reduced vagility.

## Introduction

In cooperatively breeding species of birds, grown offspring typically delay dispersal and remain at the nest to help raise non-descendant offspring [1]. The role of dispersal distance in the evolution of cooperative breeding has been the subject of debate for decades [2]. Hamilton [3], [4], for example, proposed that kin selection and altruism arose in species with delayed dispersal and short dispersal distances because these factors contributed to higher levels of interaction among kin and thus a greater opportunity for kin selection to evolve. More recently, Arnold and Owens [5] suggested that a combination of ‘slow’ life history processes and reduced dispersal are central to the evolution of cooperative breeding in birds, a conclusion based in part on prior findings such as that of Zack [6], who compared closely-related bird species and found that cooperative species dispersed less and were more philopatric than species that were not cooperative breeders. Zack went on to argue that short-distance dispersal is the result of delayed dispersal, and that non-breeding helpers are waiting to disperse to nearby territories that are of higher quality. Such individuals were hypothesized to prefer territories that are closer to their natal nest sites because they have a competitive advantage when the opportunity for dispersal to those territories arises. Under Zack's scenario, delayed dispersal and short-distance dispersal go hand-in-hand.

Evidence thus supports the hypothesis that the frequency of philopatry and short-distance dispersal is relatively high in cooperative breeders. It does not necessarily follow, however, that cooperative breeders experience fewer or shorter long-distance dispersal events, the metric likely to be more important in terms of speciation and diversification rates [7]. Indeed, recent studies on cooperative species including the Acorn Woodpecker (Melanerpes formicivorus) [8] and the Red-cockaded Woodpecker (Picoides borealis) [9] have detected significant numbers of long-distance dispersal events, indicating that the extent of long-distance dispersal may be much greater than the degree of philopatry might suggest. Part of this difference may be attributable to the inherent bias in most studies of dispersal toward short distances combined with the difficulty of detecting dispersal events beyond the limits of a study area [10]. These findings nonetheless call into question the generality of the hypothesis that cooperative breeders are characterized by reduced long-distance, as well as elevated short-distance, dispersal.

Here we conduct a comparative analysis testing the hypothesis that cooperative breeders engage in relatively fewer or shorter long-distance dispersal events than non-cooperative breeders. We do this by focusing on vagrant records—records of individuals detected outside their normal geographic range—of North American cooperatively breeding species of birds and matched non-cooperatively breeding species. To our knowledge, vagrant records have not been used previously as a measure of long-distance dispersal, although Veit [11] used vagrant sightings as a metric for population expansion. While compilations of vagrant reports do not represent complete or unbiased records of dispersal events, they are currently the only available source of information for the comparison of large-scale movements across species.

## Methods

We compiled vagrant records of seven species of cooperatively breeding birds paired with six species of non-cooperatively breeding birds (Table 1). Each cooperative species was paired as closely as possible, with reference to phylogenetic relatedness, with a non-cooperative species. In the pairings, we also tried to match species based on their migratory behavior and, when there was more than one possible match, scope of geographic range. We avoided species whose ranges covered all or much of continental North America, as such species provided little opportunity for the detection of vagrants outside their normal range. Cooperatively breeding species for which there was no well-justified non-cooperative pairing were not included in the study; these included the jays (genus *Aphelocoma*) and the nuthatches (genus *Sitta*). In the case of the anis (genus *Crotophaga*), records were combined for the two cooperatively breeding species for comparison with the single non-cooperative species.

**Table 1:** Results of paired Wilcoxon signed-rank tests.

Paired species		All records		Records >100 km	
Cooperative breeder	Non-cooperative breeder	*P*-value	*N*	*P*-value	*N*
Harris' Hawk (*Parabuteo unicinctus*)	White-tailed Hawk (*Buteo albicaudatus*)	ns	81, 15	ns	70, 2
**Groove-billed Ani (** *Crotophaga sulcirostris*) and **Smooth-billed Ani** (*C. ani*)	Mangrove Cuckoo (*Coccyzus minor*)	<0.01	55, 14	<0.01	46, 7
**Acorn Woodpecker** (*Melanerpes formicivorus*)	White-headed Woodpecker (*Picoides albolarvatus*)	<0.01	121, 19	ns	68, 14
Red-cockaded Woodpecker (*Picoides borealis*)	Ladder-backed Woodpecker (*Picoides scalaris*)	ns	4, 20	ns	1, 12
Bushtit (*Psaltriparus minimus*)	Verdin (*Auriparus flaviceps*)	ns	59, 13	ns	21, 4
Western Bluebird (*Sialia mexicana*)	**Mountain Bluebird** *(Sialia currucoides*)	<0.01	54, 163	<0.01	29, 128
Breeding range only	** Breeding range only**	<0.01	22, 56	<0.01	9, 43
Winter range only	** Winter range only**	<0.01	44, 131	<0.01	33, 97

Boldface indicates the taxon or taxa with significantly farther vagrant distances than their paired species. Sample sizes (*N*) are ordered as: cooperative breeding species, non-cooperative species. ns = *P*>0.05.

Vagrant records, defined as birds recorded outside their expected geographic range, were compiled from the regional reports published in North American Birds (formerly *American Birds*) for a ten-year period that included fall 1998 to summer 2007, inclusive. For each record of the species of interest, we noted the location and date. We then determined latitude and longitude coordinates using the searchable database provided by the Geographic Names Information System [12]. In some cases, locations specified for records referred to a relatively large geographic area, in which case we chose a landmark (usually a city or town) that was approximately at the center of the area to estimate the latitude and longitude. In cases in which a county was listed as the location, we used the county seat to approximate the location.

**Figure 1:**
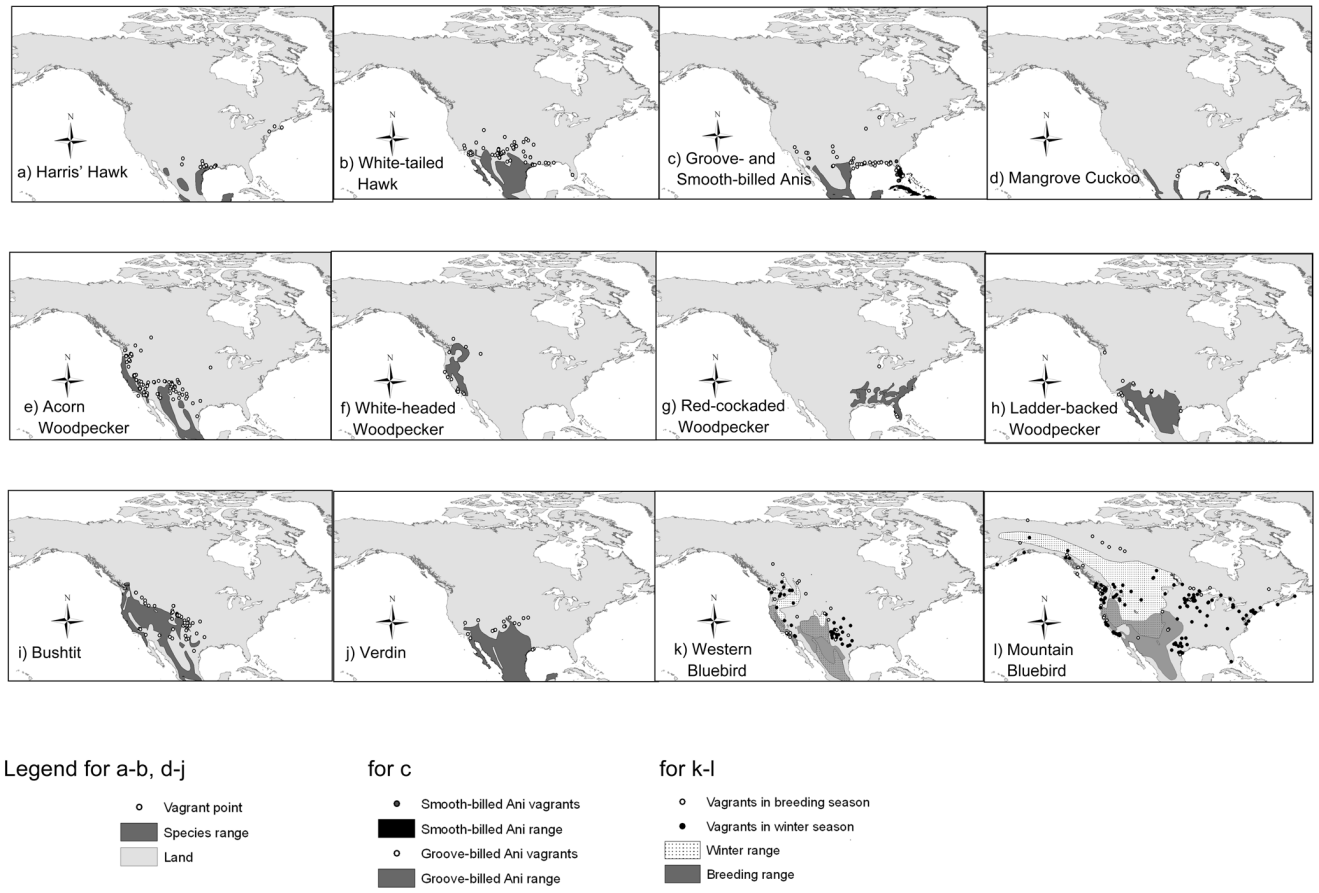
Species maps and vagrant records. Maps are presented in their original datum, North American 1983. The base map is provided by the National Geospatial-Intelligence Agency [24].

To assign species' ranges, we downloaded distribution ranges for each species as polygons from NatureServe's digital distribution maps of the birds of the Western Hemisphere [13], and imported them into ArcGIS version 10 [14]. We then plotted the coordinates for the vagrant sightings as points alongside the ranges for the corresponding species (Fig. 1).

For analysis, we included only records of birds seen outside the normal geographic ranges of the species of interest. We excluded records of notable sightings that were within the geographic range but in an unexpected habitat (e.g., urban environment or unusually high elevation).

**Figure 2:**
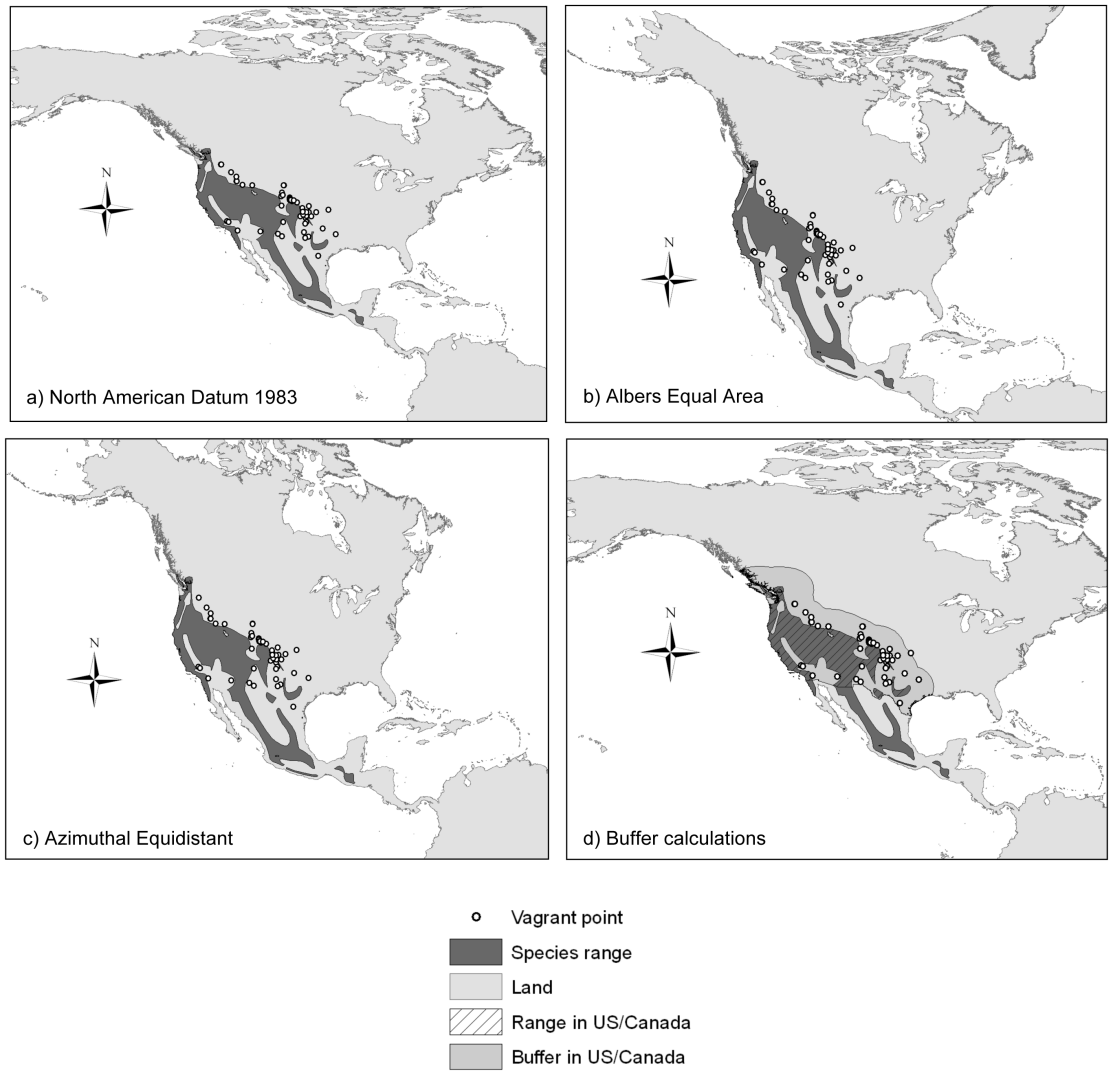
Map projections and buffer calculations, using the Bushtit (*Psaltriparus minimus*) range as an example. (a) North American 1983 is the original datum for the ranges and vagrant points. (b) Each range was reprojected to a customized Albers Equal Area projection for the purpose of calculating the centroid of the range. (c) Each range and set of vagrant points were reprojected to a customized Azimuthal Equidistant projection with the point of tangency at the centroid of the range. In this projection, the distances from the edge of the range to each vagrant point were measured. (d) A buffer at the distance from the edge of the range to the farthest vagrant was drawn around the range. The density of vagrants within the buffer was used to calculate an index to measure vagrant frequency. The base map is provided by the National Geospatial-Intelligence Agency [24].

Ranges were reprojected from their source North American 1983 datum (corresponds to WGS 1984, Fig. 2a) to an Albers Equal Area map projection that was customized for each range to minimize distortion. For each species, we found the maximum and minimum latitudes of the range and divided the area between them into six equal latitudinal sections. We defined the projection's standard parallels as the latitudes that corresponded to one-sixth from the maximum and one-sixth from the minimum of the range, according to the ‘one-sixth rule’ [15] (Fig. 2b).

In the Albers Equal Area projections, we measured the area of species ranges and calculated their centroids, defined as the geometric center of the range weighted by area. The ranges were then reprojected to customized Azimuthal Equidistant projections with the point of tangency set at the centroid of the range so that any distance measured from the center point was accurate, and distortion was minimized when measurements were made from the center outwards (Fig. 2c). Finally, distances from the closest edge of the polygon to each of the vagrant records were calculated in this map projection.

Records for each pair of species were compared using Wilcoxon signed-rank tests. The mean dispersal distance of vagrants was calculated for each species including all vagrant records and only records >100 km from the edge. The latter comparison was made to avoid bias associated with the reporting of records close to the known geographic range of a species and/or possible recent range expansion or reduction, since ranges are not necessarily static. Mean and maximum vagrancy distances were compared across all species using a paired Wilcoxon signed-rank test. Statistics were run using R [16]; alpha levels of *P*<0.05 were considered statistically significant.

In the case of the two largely migratory species included in the analyses, ranges were divided into the appropriate areas for their breeding and wintering seasons. Vagrant sightings were separated into groups based on the time of year that they were observed and were compared to the range for the corresponding season. For the Mountain Bluebird (Sialia currucoides), vagrants from October through March were compared to the wintering range, and those from April through September were compared to the breeding range [17]. Western Bluebird (Sialia mexicana) vagrants were compared to the wintering range if they were recorded from September through February, and to the breeding range from March through August [18] (Fig. 1i). We analyzed these subsets of seasonal records separately, in addition to the combined records using Wilcoxon signed-rank tests. Because the Groove-billed Ani's (Crotophaga sulcirostris) range changes only very slightly in migration, we did not analyze records with respect to season in this species.

The number of vagrants recorded for each species was compared to ensure that our results were not biased due to sample size. We deemed the number of vagrants to not be a useful comparative measure, however, because of non-congruence in geographic distribution and population size among species.

Thus, our primary focus was on the relative distances of vagrant records; that is, how far away from their normal geographic range were vagrants recorded. Only vagrant records collected in the United States and Canada were included in order to restrict records to those representing approximately equal levels of vigilance among the birding community and likelihood of publication. Records of vagrant sightings in Mexico and southward were largely unavailable or not maintained in a manner consistent with North American records and so were excluded from analysis.

In order to compare a measure of the tendency for long-distance dispersal, we also calculated an index of the relative frequency of vagrancy by the different species. Because the proportion of each species' geographic range that was within the United States and Canada varied across species, from less than 1% to 100%, the index normalized the frequency of vagrancy by dividing the number of vagrants by the area that was within the United States from a buffer drawn around the complete range polygon at the distance from the range to the farthest vagrant (Fig. 2d). This gave an estimate of the density of vagrants within the United States within the area where vagrants were likely to be reported. We also used the Partners in Flight population estimates [19] and range area calculations to estimate the densities of the species' populations. We then divided the density of vagrants by the estimated population density. Higher indices by this process correspond to higher frequencies of vagrants, taking into account the population size, range size, and geographical location of the range relative to the geographical limits of the study. This index also took population density within the range into account, a potentially important analysis to the extent that density might influence the number of vagrants per species. These indices were used as a metric for the tendency to be vagrant, and were compared pairwise across species using a Wilcoxon signed-rank test.

## Results

The number of vagrant records per species varied from 4 to 163, with a mean of 54 per taxon (Table 1). Restricting records to those >100 km from their normal geographic range, records varied from 1 to 128 with a mean of 37 per taxon. There was no significant difference in the number of records found for cooperative vs. non-cooperative breeders (all records: paired Wilcoxon signed-rank test, *P* = 0.9; records >100 kms: *P* = 1). Thus, the number of records we analyzed was comparable for cooperative and non-cooperative breeding species.

Of the six overall paired comparisons, only the one between the two bluebird species supported the hypothesis that non-cooperative species exhibit greater vagrancy than cooperative species (Table 1). In contrast, the cooperative species exhibited greater vagrancy than the non-cooperative species in two comparisons. We found no significant differences in the other three comparisons.

For the bluebird species, which are mostly migratory, additional comparisons were made by time of year. These seasonal results also showed greater vagrancy in the non-cooperative species, similar to the results from the comparison of all vagrants combined.

Three comparisons were made across all pairs of species combined. First, we tested the mean distance of vagrants from the edge of the range. This test was repeated for all vagrants and for those records >100 km. Neither comparison was statistically significant (Wilcoxon signed-rank test, *P*>0.05); that is, there was no detectable difference between cooperative and non-cooperative species in their mean vagrant distances. Second, we compared the maximum vagrant distances across all pairs of species; the difference was not significant (*P* = 0.7). Third, we compared the ratio of the density of vagrants in the U.S. and Canada to their estimated population densities within their normal ranges. Again, there was no significant difference between the cooperative and non-cooperative species (P = 0.5).

## Discussion

Short-distance dispersal and natal philopatry have been central to the discussion of cooperative breeding and helping behavior, both in terms of their evolutionary origins and their adaptive significance [2], [5], [20]. What seems like a logical relationship between short-distance dispersal and cooperation may, however, be more complicated than is often assumed. For example, in addition to facilitating cooperation, limited dispersal has the potential to increase the probability of incest [21] and competition among kin that are in close proximity to one another [22]. In addition, a relatively high frequency of philopatry and short-distance dispersal may or may not translate into a reduction in long-distance dispersal events that are more likely to extend the geographic range of species and ultimately lead to increased diversification and speciation [7].

In this study we focused on vagrant records as a measure of long-distance dispersal. Observers throughout North America note and report vagrants, and these observations are scrutinized and published in *North* American Birds. While these observations likely represent a small proportion of vagrants for any of the species, such records are most likely unbiased with respect to the species pairs used here.

One source of these vagrants is presumably females that have been observed to engage in long distance dispersal events in several species. Zack [6] recognized that some non-breeding birds in cooperative groups might attempt to disperse longer distances after an unsuccessful period of waiting for a breeding opportunity to arise nearby. In his compilation of dispersal distances, however, few of the cooperative species were recorded as traveling farther than six territories away from the natal nest site, and even those events were relatively rare. Long-distance dispersal clearly occurs, however. For example, Acorn Woodpeckers have extended their range and colonized several islands and areas 30–200 km from established populations within historic times [23], and one of us (ELW) recorded a female Red-cockaded Woodpecker that dispersed 322 km from Georgia to Florida. Events such as these are unlikely to be detected in standard field studies due to the inevitable bias associated with difficulty of detecting movements of birds away from a study area [10]. Additionally, because the female Red-cockaded Woodpecker dispersed within the range of the species, she would not have been noticed as a vagrant using the methods of reporting in *North American Birds*. Dispersal events of this magnitude probably occur much more frequently than current detection methods indicate.

Defining a vagrant can be problematic. For the purpose of calculating mean distances from the edge of the range, we used two tiers of measurement. First, we included all records that were outside of the range for each species. In some cases, these records could potentially be only a few kilometers from the normal geographic range. While these distances might not seem to be large enough to draw any conclusions, the fact that they were reported in *North American Birds* suggests that they were important enough to include in this study. A potentially larger problem is the rigid definition of geographic range, because in many cases, ranges are not static. Our analysis of vagrants detected >100 km from their normal geographic range attempts to take this problem into consideration, but is still unlikely to be perfect in all cases.

The map projections were another challenge, because there is no steadfastly reliable method for measuring long distances and large areas on a continent-wide scale without distortion. To minimize this problem, we created custom projections for all of the ranges rather than using a single projection for all of North America. The custom-made projections were specific to the geographic parameters of each range to minimize the distortion for each species. Thus, although some distortion was unavoidable, this is unlikely to have biased our results in any systematic way.

Overall, we found no significant difference in the frequency of vagrants or the mean or maximum distance that vagrants traveled outside their normal range. In the individual comparisons of distance, there was no significant difference in three of the pairs. In two of the pairings, the cooperative species had longer vagrant distances in at least one of the tests, while in only one pair did the non-cooperative species exhibit significantly longer vagrant distances than the cooperatively breeding species.

In summary, our results fail to support the hypothesis that cooperative breeders engage in significantly fewer or shorter long-distance dispersal events than non-cooperative breeders. The abundance of vagrants in cooperatively breeding species of birds might be representative of ‘fat-tailed’ dispersal distributions, such as that recorded for Red-cockaded Woodpeckers [9], in which the number of dispersal events trails off at a slow rate with distance from the nest, with relatively frequent observations at greater distances from the natal territory. In any case, the relative vagility among cooperative breeders found in this study fails to support the hypothesis that the reduced rate of speciation among cooperative breeders indicated by Cockburn's [7] analysis is a consequence of these species being relatively poor colonists compared to non-cooperative species. Instead, as pointed out by Cockburn, most of the apparent difference in diversification rates is more likely to be due to the lower frequency of migratory behavior observed in non-cooperative taxa.

Published sightings of vagrants likely only represent a small proportion of individuals that disperse outside of their typical geographic ranges. While there are likely some inherent biases in the degree to which vagrants are sighted and recorded, we have no reason to believe that such biases would systematically involve cooperative species. Thus, while the vagrant records in this study do not represent an exhaustive summary of all long-distance dispersal events, they provide a useful means of comparison between the broader groupings of species (cooperative and non-cooperative).

Measuring long-distance dispersal events will undoubtedly be easier in the future as GPS, genetic, and other technologies become more refined for use on smaller taxa. At this point, however, the only feasible way to accomplish a comparison of this scope is to use records of vagrant sightings, a resource largely overlooked until now. Ultimately, our results demonstrate the degree to which we do not yet understand the role of dispersal in the evolution of cooperation and point to the necessity for further investigation in this field.
